# C11orf95-RELA reprograms 3D epigenome in supratentorial ependymoma

**DOI:** 10.1007/s00401-020-02225-8

**Published:** 2020-09-09

**Authors:** Jacqueline Jufen Zhu, Nathaniel Jillette, Xiao-Nan Li, Albert Wu Cheng, Ching C. Lau

**Affiliations:** 1 The Jackson Laboratory for Genomic Medicine, Farmington, CT USA; 2 Texas Children’s Cancer Center, Baylor College of Medicine, Houston, TX USA; 3 Department of Pediatrics, Northwestern University, Chicago, IL USA; 4 The Jackson Laboratory Cancer Center, Bar Harbor, ME USA; 5 Department of Genetics and Genome Sciences, University of Connecticut Health Center, Farmington, CT USA; 6 Institute for Systems Genomics, University of Connecticut Health Center, Farmington, CT USA; 7 Division of Hematology-Oncology, Connecticut Children’s Medical Center, Hartford, CT USA; 8 Department of Pediatrics, University of Connecticut Health Center, Farmington, CT USA

**Keywords:** C11orf95-RELA, Supratentorial ependymoma, Transcription factor, 3D genome

## Abstract

Supratentorial ependymoma (ST-EPN) is a type of malignant brain tumor mainly seen in children. Since 2014, it has been known that an intrachromosomal fusion *C11orf95-RELA* is an oncogenic driver in ST-EPN [Parker et al. Nature 506:451–455 (2014); Pietsch et al. Acta Neuropathol 127:609–611 (2014)] but the molecular mechanisms of oncogenesis are unclear. Here we show that the C11orf95 component of the fusion protein dictates DNA binding activity while the RELA component is required for driving the expression of ependymoma-associated genes. Epigenomic characterizations using ChIP-seq and HiChIP approaches reveal that C11orf95-RELA modulates chromatin states and mediates chromatin interactions, leading to transcriptional reprogramming in ependymoma cells. Our findings provide important characterization of the molecular underpinning of *C11orf95-RELA* fusion and shed light on potential therapeutic targets for *C11orf95-RELA* subtype ependymoma.

**Electronic supplementary material:**

The online version of this article (10.1007/s00401-020-02225-8) contains supplementary material, which is available to authorized users.

## Introduction

Ependymoma is the third most common malignant brain tumor in children. It can be classified into three groups based on location of the tumor: supratentorial, infratentorial and spinal. Currently there is no effective chemotherapy identified and treatment is limited to surgery with or without adjuvant radiation therapy. Because a significant portion of ependymoma is found in young children, radiation therapy is not desirable due to the detrimental effects on the developing brain. Thus, there is an urgent need to develop targeted therapy based on the underlying biology. *C11orf95-RELA* fusion was found to be the most recurrent structural variation in approximately 70% of supratentorial ependymomas (ST-EPN) [14, 15]. Neural stem cells transformed by C11orf95-RELA were able to form brain tumor in mice [11, 14]. However, how C11orf95-RELA functions in tumorigenesis at the molecular level is largely unknown. We present results here showing that contrary to the hypothesis that C11orf95-RELA ependymoma is driven by the RELA component of the fusion, binding affinity of the fusion protein to DNA is dictated by the C11orf95 component. The contribution of the RELA component is to stabilize the DNA binding of the fusion protein and provide its activation domain to drive expression of the target genes.

## Materials and methods

### Cell culture

HEK293T and its derived G16-2, G16-3, G16-4 cells as well as BXD-1425-EPN cells were cultured in Dulbecco’s modified Eagle’s medium (Sigma) with 10% fetal bovine serum (Lonza), 4% Glutamax (Gibco), 1% sodium pyruvate (Gibco) and penicillin–streptomycin (Gibco). Incubator conditions were 37 °C and 5% CO_2_. Protein overexpression in G16-2, G16-3 and G16-4 cells were induced by 1 μg/ml doxycycline treatment for 2 days.

### Virus production

A viral packaging mix of pLP1, pLP2, and VSV-G were co-transfected with each lentiviral vector (pCW-RELA-HA, pCW-C11orf95^fus1^-HA, or pCW-C11orf95-RELA^fus1^-HA) into Lenti-X 293T cells (Clontech), seeded the day before in 6-well plates at a concentration of 1.2 × 10^6^ cells per well, using Lipofectamine 3000 (ThermoFisher). Media was changed 6 h after transfection then incubated overnight. Twenty-eight hours post transfection, the media supernatant containing virus was filtered using 45 μm PES filters then stored at − 80 °C until use.

### Cell line generation

To make G16-2, G16-3, and G16-4 cell lines, the day prior to transduction, HEK293T cells were seeded into 12-well plates at a density of 1.5 × 10^5^ cells per well. Prior to transduction, media was changed to that containing 10 μg/ml polybrene, 1 ml per well. 250 μl of each respective virus was added to each well and incubated overnight. Media was changed 24 h post-transduction. Four days post-transduction, cells were split. Five days post-transduction, media with antibiotics (2 μg/ml Puromycin) was added to each respective well of one replicate plate. Antibiotic selection continued for at least 2 weeks before use in downstream experiments.

### Cloning

ORF encoding C11orf95^fus1^ was obtained as IDT gBlock while HEK293T cDNA served as template for amplifying RELA sequence. PCR and SLIC cloning were used to insert ORF encoding C11orf95^fus1^, C11orf95-RELA^fus1^, RELA with C-terminal HA tag into Gateway donor vector (pCR8/GW/TOPO, Invitrogen). Then LR clonase II reactions were used to shuttle ORFs into pCW-DEST (lentiviral Dox-inducible expression) derived from pCW-Cas9 (Addgene # 50661), and pmax-DEST (transient constitutive expression, Addgene # 48222) vectors, generating pCW-C11orf95^fus1^-HA, or pCW-C11orf95-RELA^fus1^-HA, pCW-RELA-HA lentiviral Dox-inducible plasmids and pmax-C11orf95^fus1^-HA, pmax-C11orf95-RELA^fus1^-HA, pmax-RELA-HA transient expression plasmids. pmax-Clover and pmax-C11orf95-Clover, and pmax-C11orf95^fus1^-VP64, pmax-mScarlet-H2A, plasmids were generated with a combination of PCR, restriction-ligation, SLIC and LR Clonase II reactions. GFP-reporter plasmids, 15xUSF1-minCMV-EGFP and 15xC11orf95-minCMV-EGFP, were generated by ligation of annealed oligos containing USF1 or C11orf95 binding sites into minCMV-EGFP plasmid with non-palindromic overhang sites via digestion with BsaI, subsequently screened for the number of binding sites by Sanger sequencing.

### Transfection and imaging

Cells were seeded into 12-well plates at a density of 1.5 × 10^5^ cells per well the day before transfection. For imaging experiments, 300 ng of pmax-C11orf95^fus1^-Clover or pmax-Clover (unfused control) was co-transfected with 100 ng of pmax-mScarlet-H2A (nuclear marker) using 1.5 µL Attractene transfection reagent (Qiagen). Microscopy images were taken with the iRiS Digital Cell Imaging System (Logos Biosystems).

### Flow cytometry

GFP-reporter plasmid DNA (300 ng) containing 15xUSF1 binding sites (15xUSF1-minCMV-EGFP) or 15xC11orf95 binding sites (15xC11orf95-minCMV-EGFP) was co-transfected with 300 ng of plasmid expressing test transcription factor constructs (EmptyVector, pmax-C11orf95^fus1^, pmax-C11orf95-RELA^fus1^, pmax-RELA, pmax-C11orf95^fus1^-VP64) using 1.5 µl Lipofectamine 3000 (ThermoFisher). 48 h after transfection, cells were trypsinized, suspended in media then analyzed on a LSRFortessa X-20 flow cytometer (BD Bioscience). Fifty thousand events were collected each run.

### Western blot

Western blot was performed using general protocol from Bio-Rad. Primary antibodies used were HA antibody (Cell Signaling Technology 3724S), RELA antibody (Abcam ab32536), and β-actin antibody (Cell Signaling Technology 4970S).

### Genotyping

BXD-1425-EPN DNA was extracted using DNeasy Blood & Tissue Kit (Qiagen), followed by genotyping PCR using Phusion PCR protocol (NEB) with primer pair of forward: CCTGCACCTGGACGACAT and reverse: TTGGTGGTATCTGTGCTCCTC. PCR amplicons were sent for Sanger sequencing, and analyzed by ApE.

### Gene ontology analysis

Gene ontology analysis was done with Metascape (https://metascape.org/).

### DNA motif analysis

ChIP-seq peak sequences were uploaded and analyzed by MEME-ChIP (https://meme-suite.org/tools/meme-chip). Motifs were found de novo by DREAM. Sequences that had specific motif were reported by FIMO.

### RNA-seq and analysis

RNA was extracted using RNeasy Mini Kit (Qiagen), then sent to Genewiz for library construction and sequencing by Illumina Nextseq sequencer. Each sample had two biological replicates. RNA-seq data was quantified using Salmon (version 0.8.0), followed by differential expression analysis using DEseq2. The cutoff for identifying differentially expressed genes were set as FDR < 0.05 and fold change ≥ 1.5.

### ChIP-seq and analysis

ChIP was performed using iDeal ChIP-seq kit for Transcription Factors (Diagenode) and protocol provided. HA (Cell Signaling Technology 3724S), RELA (Abcam ab32536) and H3K27ac (Cell Signaling Technology 8173S) antibodies were used for chromatin pull-down. Libraries were constructed using KAPA Hyper Prep Kits (Roche) and NEBNext® Multiplex Oligos for Illumina® (NEB) for Illumina Nextseq sequencing. Each sample had two biological replicates. The reproducibility between replicates was evaluated by Pearson correlation coefficient of read counts using deepTools (version 3.3.0) which was all greater than 0.95. Replicates were then combined for peak calling.

Sequencing data was mapped to hg38 reference genome by Bowtie2 (version 2.3.1). Sequences with mapping quality score greater than ten were accepted for the following analysis. Peaks were called by MACS2 (version 2.1.0.20151222) using “–q 0.01 –nomodel –extsize 200” parameter for transcription factor ChIP-seq, and using “–broad −q 0.05, –nomodel –extsize 220” for histone mark ChIP-seq. For peaks that had at least 1 bp overlap, we considered them as overlapped peaks. The peak was considered to locate at gene promoter when it had at least 1 bp overlap with the promoter region. Promoter region was defined as a 5 kb span extending 2.5 kb at both sides from the transcription start site (TSS). Differential H3K27ac peaks were called by MACS2 bdgdiff subcommand. Read density heatmap of ChIP-seq peaks were computed and plotted using deepTools (version 3.3.0). Data was viewed by Integrative Genomics Viewer (IGV).

### HiChIP and analysis

HiChIP was performed following protocol published by Mumbach et al. 2017, with minor modifications. HA (Cell Signaling Technology 3724S) and H3K27ac (Cell Signaling Technology 8173S) antibodies were used for chromatin pull-down. Libraries were constructed using KAPA Hyper Prep Kits (Roche) and NEBNext® Multiplex Oligos for Illumina® (NEB) for Illumina Novaseq sequencing. Each sample had two biological replicates. Replicate data was combined for analysis. Raw data was processed by HiC-Pro [17] using hg38 reference genome. Chromatin interactions were called using hichipper [6] by providing certain ChIP-seq peaks and HiC-Pro outputs. Interaction heatmap was plotted by HiCPlotter [1] at 10 kb resolution. Interactions associated with specific genes were filtered by interaction count greater than one, then visualized on WashU Epigenome Browser, or processed for enhancer-promoter analysis.

### Canonical pathway analysis

Canonical pathway analysis was done by Ingenuity Pathway Analysis (IPA) using “Core Analysis” function.

## Results

### C11orf95-RELA is a novel transcription factor that recognizes a specific DNA motif dictated by the C11orf95 fragment

**Fig. 1:**
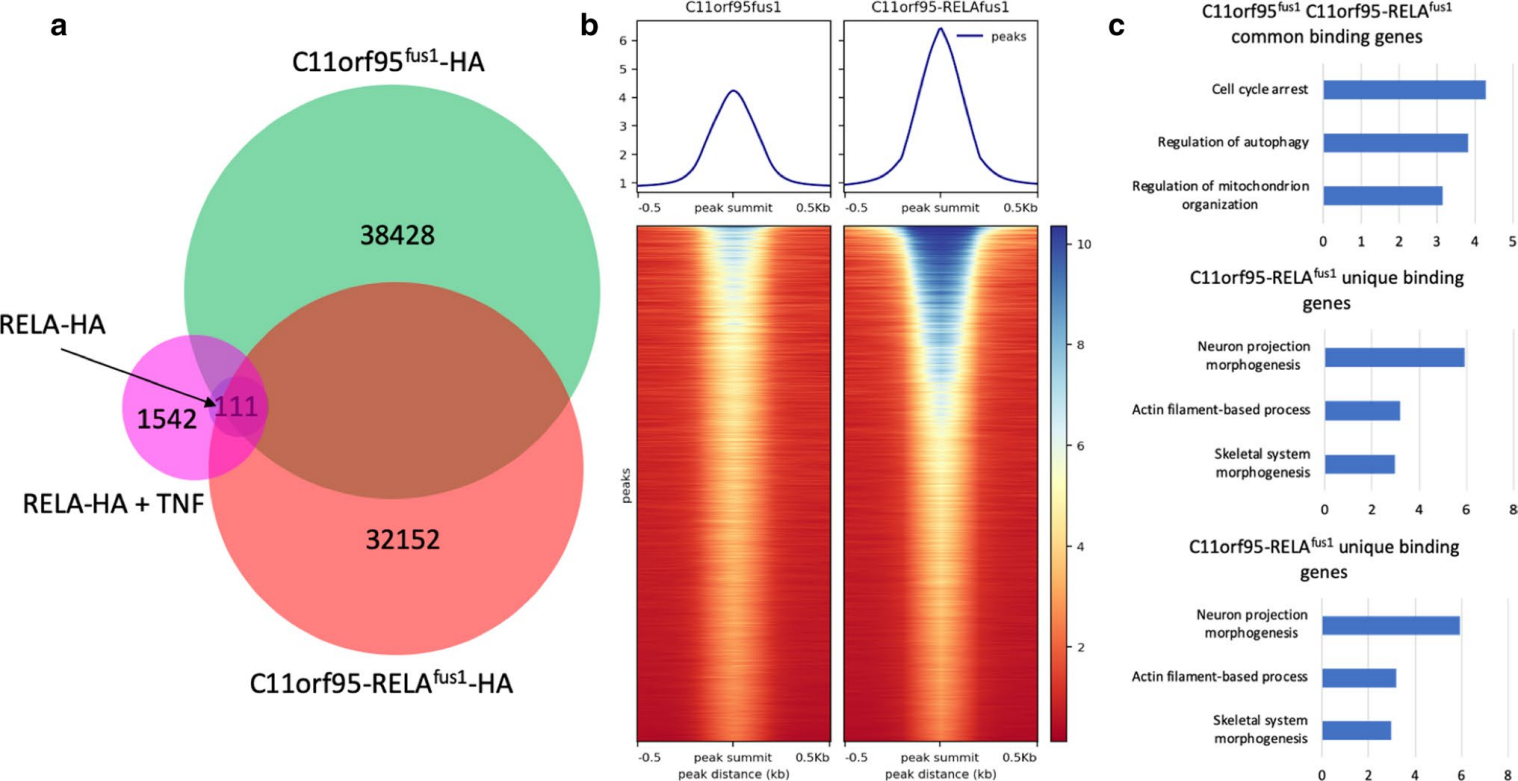
**a** Venn diagram of ChIP-seq peaks. The DNA pull-down were done by RELA-HA (blue, the smallest circle) in G16-2 cells, RELA-HA after TNF treatment (magenta) in G16-2 cells, C11orf95^fus1^-HA (green) in G16-3 cells and C11orf95-RELA^fus1^-HA (red) in G16-4 cells. The numbers indicate the total peaks identified in each cohort. The Venn diagram is not dawn to scale. **b** Read densities over ChIP-seq peaks of C11orf95^fus1^ and C11orf95-RELA^fus1^. Using peak summit as the center, the normalized mapped reads over a 1 kb region around the peak were plotted for a total of 16,829 shared peaks of C11orf95^fus1^ and C11orf95-RELA^fus1^. **c** Gene ontology analysis on C11orf95^fus1^ and C11orf95-RELA^fus1^ common binding genes, C11orf95^fus1^ unique binding genes and C11orf95-RELA^fus1^ unique binding genes. The top three ranked biological processes according to *q* value are shown

*C11orf95-RELA*^*fus1*^ was identified as the most frequently occurring fusion subtype in supratentorial ependymoma with *C11orf95-RELA* fusion (ST-EPN-RELA), containing almost the entire *RELA* gene except the first two codons fused to a truncated *C11orf95* gene fragment (*C11orf95*^*fus1*^) harboring two and a half exons out of five exons of the full-size gene [14]. Unlike RELA, which is a well-known transcription factor subunit of NF-κB, C11orf95 is an uncharacterized protein. While nuclear localization of RELA is dependent on NF-κB activation, C11orf95-RELA fusion protein is constitutively localized in the nucleus in transformed HEK293T and mouse neural stem cells [14]. Intriguingly, *C11orf95*^*fus1*^ contains a C_2_H_2_ zinc finger domain typically found in transcription factors (Supplementary Fig. 1, online resource), implying its DNA binding potential. We hypothesized that the fusion of C11orf95 and RELA create a novel transcription factor whose DNA binding affinity as well as nuclear localization are largely governed by the C11orf95 fragment. Interestingly, fusion of C11orf95^fus1^ fragment to GFP is sufficient to drive exclusive localization of the fluorescence signal in the nucleus of HEK293T cells, demonstrating the intrinsic propensity of the C11orf95 ^fus1^ fragment to translocate into the nucleus (Supplementary Fig. 2, online resource). However, there was previously no direct evidence for the binding of C11orf95^fus1^ and C11orf95-RELA^fus1^ to DNA. To address this question and further decipher the regulatory mechanisms of the fusion protein, we engineered HEK293T cell lines to express C11orf95-RELA^fus1^ (G16-4), C11orf95^fus1^ fragment (G16-3), or the RELA fragment (G16-2) in order to study the contribution of the fusion protein and each of the partners to epigenetic program and transcriptional regulation. These transgenes are tagged with the HA-epitope and are under the control of a doxycycline-inducible TetO promoter (Supplementary Fig. 3a, b, online resource). The HA tag facilitates our molecular experimentations such as Western blot and ChIP, despite the lack of good quality antibodies against the fusion and C11orf95 proteins. We then conducted ChIP-seq using HA antibody to profile genome-wide chromatin bindings of RELA, C11orf95^fus1^ and C11orf95-RELA^fus1^ in G16-2, G16-3 and G16-4 cells, respectively. Surprisingly, 32,152 and 38,428 binding peaks were identified for C11orf95-RELA^fus1^ and C11orf95^fus1^, respectively, with 16,829 overlapping (Fig. 1a). In contrast, RELA ChIP-seq only identified 111 binding sites in total. Considering RELA is normally inactive without cellular stimulation, we treated G16-2 cells with tumor necrosis factor (TNF) for six hours after doxycycline induction of RELA expression, in order to capture RELA bindings in its active state. As a result, 1542 binding peaks were identified, which was still far fewer than the binding peaks identified for the fusion or C11orf95. Among the RELA binding peaks identified in the presence of TNF stimulation, less than 25% overlapped with C11orf95-RELA^fus1^ peaks (Fig. 1a). These results indicate that C11orf95^fus1^ contributes to the majority of DNA binding of the fusion protein.

Although both C11orf95^fus1^ and C11orf95-RELA^fus1^ can bind to DNA, their binding targets were not completely identical. Other than the common peaks, there were also specific peaks for each of the two proteins (Fig. 1a). Among the 16,829 shared peaks, C11orf95-RELA^fus1^ had significantly higher signal densities than C11orf95^fus1^ after sequencing read normalization (Fig. 1b), suggesting the RELA portion of the fusion protein might stabilize DNA binding. Furthermore, we selected genes that were bound by C11orf95^fus1^ and/or C11orf95-RELA^fus1^ at promoter regions, categorized them into three groups: C11orf95^fus1^ and C11orf95-RELA^fus1^ common binding genes, C11orf95^fus1^ unique binding genes and C11orf95-RELA^fus1^ unique binding genes. Gene ontology (GO) analysis showed C11orf95-RELA^fus1^ unique binding genes were specifically enriched in neuron projection morphogenesis, actin filament process and skeletal system morphogenesis, which are known to be associated with brain or brain disease development [3, 7, 16, 18]. C11orf95^fus1^ and C11orf95-RELA^fus1^ common binding genes were enriched in cell cycle arrest, regulation of autophagy and regulation of mitochondrion organization. However, C11orf95^fus1^ unique binding genes were enriched in more fundamental biological processes such as translation, carbohydrate derivative biosynthetic process and asparagine N-linked glycosylation (Fig. 1c, Supplementary Table 1, online resource). In addition, we also obtained gene expression profiles of G16-3 and G16-4, as well as HEK293T control cells by RNA-seq to interrogate the transcriptional effects of C11orf95^fus1^ and C11orf95-RELA^fus1^. Sixty-six and 210 genes were identified to be up-regulated (FDR < 0.05, fold change ≥ 1.5) in G16-3 and G16-4 cells, respectively, compared to the controls. In parallel, we compiled a list of 886 ST-EPN-RELA-associated genes (Supplementary Table 2, online resource) based on published data [13, 14, 19]. By comparing G16-3 and G16-4 up-regulated genes to ST-EPN-RELA-associated genes, only three of G16-3 (C11orf95^fus1^) up-regulated genes overlapped with ST-EPN-RELA associated genes, while 67 of G16-4 (C11orf95-RELA^fus1^) up-regulated genes overlapped the ST-EPN-RELA associated gene list, including some well-known marker genes such as *L1CAM*, *CHD5* and *NOTCH1* (Supplementary Fig. 4a, b, online resource). All these observations collectively indicate that C11orf95^fus1^ alone is not sufficient to drive transcriptomic dysregulation like C11orf95-RELA^fus1^.

**Fig. 2:**
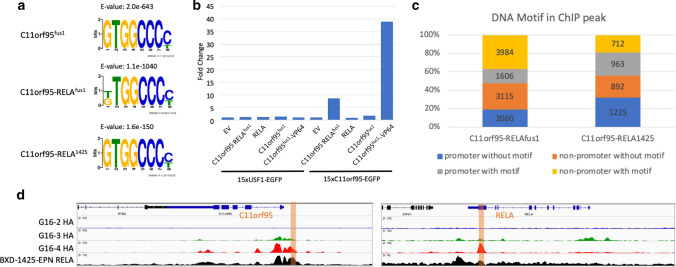
**a** DNA binding motifs enriched for C11orf95^fus1^ and C11orf95-RELA^fus1^ and C11orf95-RELA^1425^ in G16-3, G16-4 and BXD-1425-EPN cells, respectively. **b** Reporter assay. Reporter EGFP gene was linked to identified C11orf95 binding motif as well as USF1 motif as control. Empty vector (EV), C11orf95-RELA^fus1^, RELA, C11orf95^fus1^ or activation domain VP64 fused C11orf95^fus1^ were expressed to test EGFP expression. EGFP signals were measured by flow cytometry. **c** C11orf95 binding motif distribution in C11orf95-RELA peaks in G16-4 and BXD-1425-EPN cells. **d** RELA, C11orf95^fus1^, C11orf95-RELA^fus1^ and C11orf95-RELA^1425^ binding profiles at *C11orf95* and *RELA* genes. The orange bars indicate the regions that have C11orf95 binding motifs

While the HEK293T transgenic cell models allow the dissection of the molecular function of C11orf95-RELA and its constituents, to obtain insights of the epigenomic and transcriptomic effects of C11orf95-RELA in the context of ependymoma, we performed further experiments in an ependymoma cell line, BXD-1425-EPN [19] that was established from an orthotopic patient-derived xenograft (PDX) originating from a ST-EPN-RELA tumor. Genotyping-sequencing and Western blot experiments with BXD-1425-EPN identified a novel fusion configuration containing the first three exons and a small fragment of the fourth exon of *C11orf95* gene, and *RELA* without the first two and half exons (Supplementary Fig. 3c, online resource), here referred to as *C11orf95-RELA*^*1425*^. Despite the lack of fusion-specific antibody, the fact that unstimulated RELA is excluded from the nucleus [5] while the fusion protein binds to DNA allowed us to use anti-RELA antibody to conduct ChIP-seq to pull down C11orf95-RELA^1425^ bound chromatin. RELA ChIP-seq in BXD-1425-EPN cells identified 13,954 chromatin binding sites of C11orf95-RELA^1425^, with 5,338 commonly shared by C11orf95-RELA^fus1^ bindings in G16-4 cells. With the binding peak sequences of C11orf95^fus1^, C11orf95-RELA^fus1^ and C11orf-RELA^1425^, we implemented de novo motif discovery using MEME-ChIP tool [8]. An identical GC-rich motif (GTGGCCCC) was readily recovered with top scores from all three sets of ChIP-seq peak sequences (Fig. 2a), supporting the validity of fusion protein pull-down by RELA antibody in BXD-1425-EPN cells and further confirming that the C11orf95^fus1^ fragment dictates DNA binding specificity of the fusion protein. As the motif consensus was repeatedly enriched by using different cells with different types of C11orf95-RELA fusion and different antibodies, the identified C11orf95 binding specificity is highly faithful. To establish GTGGCCCC as the *bona fide* DNA binding motif of C11orf95, we performed a reporter assay with 15 copies of GTGGCCCC and a minimum promoter cloned upstream of an EGFP reporter gene (Fig. 2b). As expected, C11orf95-RELA^fus1^ activated EGFP reporter expression harboring copies of the putative C11orf95 motif. On the contrary, the unfused constituents RELA or C11orf95^fus1^ were unable to activate the reporter. Furthermore, supplementing C11orf95^fus1^ with a heterologous VP64 activation domain in the form of C11orf95^fus1^-VP64 fusion endowed transactivation activity on the reporter with 15 copies of the putative C11orf95 (GTGGCCCC) DNA binding motif but not on a control reporter harboring 15 copies of an unrelated USF1 motif. These results suggest that C11orf95^fus1^ binds to the GC-rich motif GTGGCCCC and may activate gene expression by hijacking the transcriptional activation domain of RELA, as in the case of the C11orf95-RELA fusion. Furthermore, to examine the functional region of C11orf95^fus1^, we constructed three truncation mutants of C11orf95^fus1^-VP64 fusions and performed reporter assay to measure their transactivation activities (Supplementary Fig. 5, online resource). Deletion of the zinc finger (ZF) domain led to the loss of transactivation activity. In addition, deletion of the N-terminal and C-terminal regions upstream and downstream of the ZF domain also resulted in the loss of transactivation activity (Supplementary Fig. 5, online resource), suggesting C11orf95^fus1^ might be the minimal fragment of C11orf95 required for DNA binding. These results and the observation by Parker et al. [14] that C11orf95^fus1^ is the most frequent breakpoint of C11orf95 and is contained in all identified C11orf95-RELA fusion subtypes prompted us to speculate that C11orf95^fus1^ is the minimal fragment required for a functional C11orf95-RELA fusion.

Among the top-scoring ChIP-seq peaks of C11orf95-RELA^fus1^ and C11orf95-RELA^1425^, approximately half of them contain one or more of the C11orf95 DNA binding motif (Fig. 2c), implying a direct binding by the fusion protein. The no-motif peaks might be bound by a C11orf95-RELA-containing protein complex that uses another pioneer subunit to initiate chromatin binding. Both motif and no-motif peaks were distributed at either promoter or non-promoter region with no significant bias (Fig. 2c). Interestingly, ChIP-seq experiments in G16-4 and BXD-1425-EPN cells identified fusion binding peaks at *C11orf95* promoter and *RELA* transcription end sites, with the C11orf95 DNA binding motif observed in both peaks (Fig. 2d), suggesting the possibility of auto-regulation. To evaluate such hypothesis, we determined whether expression of endogenous *C11orf95* in G16-4 cells was induced by exogenous C11orf95-RELA^fus1^ provided by the transgene. The alternative codon usage adopted in the *C11orf95*^*fus1*^ transgene construction allowed us to encode the same C11orf95^fus1^ protein sequence with synonymous codons for distinguishing sequencing reads derived from transgenic and endogenous *C11orf95* in RNA-seq experiments (Supplementary Fig. 1, online resource). A two-fold up-regulation of endogenous *C11orf95* transcripts was observed in G16-4 but not in G16-3 cells after doxycycline induction, validating the potential of C11orf95-RELA to positively auto-regulate its expression via binding to the *C11orf95* promoter (Supplementary Fig. 4b, online resource).

### C11orf95-RELA modulates chromatin state and mediates chromatin interaction

**Fig. 3:**
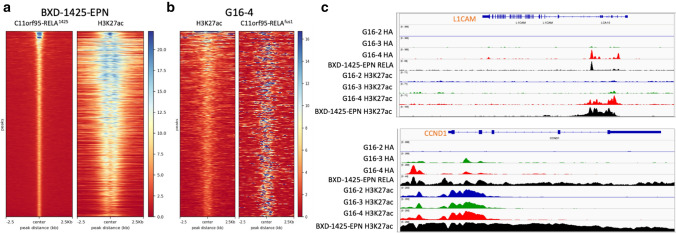
**a** ChIP-seq read densities of C11orf95-RELA^1425^ and H3K27ac in BXD-1425-EPN cells. 3792 top-scoring C11orf94-RELA^1425^ ChIP-seq peaks were used to plot the heatmap. The left side heatmap showed C11orf95-RELA^1425^ signals along a 5 kb region centering the peak summit. The right side heatmap showed H3K27ac signals along the same regions. **b** Read densities of H3K27ac and C11orf95-RELA^fus1^ in G16-4 cells. 441 differential H3K27ac peaks in G16-4 compared to G16-2 cells were used to plot the heatmap. The left side heatmap showed H3K27ac signals along a 5 kb region centered with H3K27ac peak center. The right side heatmap showed C11orf95-RELA^fus1^ signals along the same regions. **c** RELA, C11orf95^fus1^, C11orf95-RELA^fus1^ and C11orf95-RELA^1425^ as well as corresponding H3K27ac ChIP-seq profiles at ST-EPN-RELA marker genes *CCND1* and *L1CAM*

We applied H3K27ac ChIP-seq to profile active chromatin regions in HEK293T transgenic cell models and BXD-1425-EPN to address the correlation between the fusion protein binding and chromatin state. At 3,792 fusion protein binding sites with top confidence in BXD-1425-EPN cells, strong H3K27ac signals were observed (Fig. 3a). Comparative analysis of genome-wide H3K27ac profiles between transgenic HEK293T cell models expressing fusion (G16-4) and RELA (G16-2) identified 441 differential H3K27ac peaks specific in G16-4 cells. These G16-4 specific H3K27ac peaks highly correlated with fusion protein binding (Fig. 3b) with over 90% of them overlapping with fusion protein binding sites, implying the C11orf95-RELA was able to initiate chromatin state changes. *CCND1* and *L1CAM*, two well-known marker genes of ST-EPN-RELA, exemplify two different chromatin state outcomes upon fusion protein binding. At *L1CAM* promoter region, fusion protein binding likely resulted in the deposition of H3K27ac marks (Fig. 3c). On the contrary, *CCND1* promoter retained its active chromatin state regardless of the presence (in G16-4 and BXD-1425-EPN cells) or absence (in G16-2 and G16-3 cells) of the fusion protein binding (Fig. 3c).

**Fig. 4:**
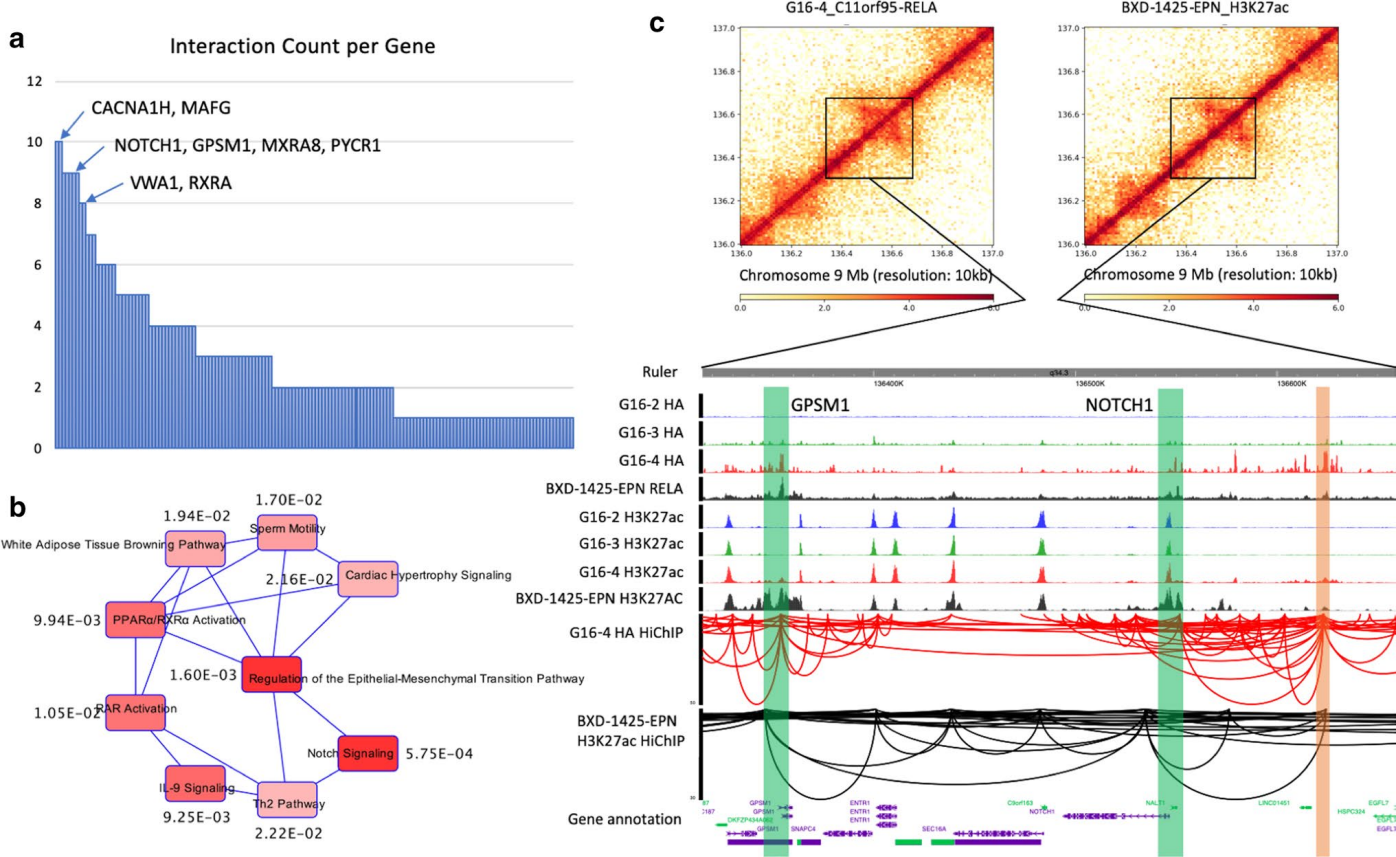
**a** Histogram of interaction counts per gene. The interactions coming out from the 156 identified gene promoters were counted. **b** Canonical pathway analysis of 102 genes that had more than one interaction. The numbers by the pathways are *p* values. **c** Chromatin interactions captured by HiChIP along *NOTCH1* and *GPSM1* genes. The heatmaps showed a 1 Mb region of interactions along *NOTCH1* and *GPSM1* genes. The browser view showed the zoom-in details of the black box indicating region in the heatmaps, including ChIP-seq profiles and HiChIP interactions. The green bars indicate *NOTCH1* and *GPSM1* promoters. The orange bar indicates the enhancer

As three-dimensional chromatin structure is known to be tightly associated with disease and cancer development, we next sought to identify changes in genome structure mediated by the fusion in ST-EPN-RELA. We utilized a sequencing-based chromatin capture approach HiC Chromatin Immunoprecipitation (HiChIP) [10] to characterize genome-wide chromatin interactions and connect the protein binding peaks into loops and networks. We used anti-HA antibody to perform HiChIP of HA-tagged fusion protein in G16-4 cell lines. By further filtering with C11orf95-RELA^fus1^-HA ChIP-seq peaks, we identified a total of 32,669 high-confidence interactions. For BXD-1425-EPN cells, we performed H3K27ac HiChIP and identified 15,777 C11orf95-RELA^1425^ mediated interactions filtered by both H3K27ac and RELA ChIP-seq peaks. We mapped HiChIP identified chromatin interactions to the promoter regions of 886 ST-EPN-RELA associated genes mentioned above (Supplementary Table 2, online resource) to identify ST-EPN-RELA gene associated interactions. As a result, a total of 445 common interactions were identified in both G16-4 and BXD-1425-EPN cells. Interestingly, not all the 886 genes harbored chromatin interaction(s) to other locus/loci, but only 156 of them (Supplementary Table 3, online resource) reached out to one or more promoter or non-promoter (enhancer) regions. Since these ST-EPN-RELA gene associated chromatin interactions were directly mediated by the fusion protein, it is reasonable to speculate that the more interacting partners one gene has, the more regulatory events it is involved in, and the more important roles it plays in ST-EPN-RELA. In other words, the genes that have more interactions with other genomic loci are more likely to be the hub of the fusion protein mediated network and more delicately regulated in fusion-associated transcription, so that they might contribute more to ependymoma development. Hence, we counted the number of interactions each gene promoter is involved in and plotted a histogram ranking the 156 genes by their interaction counts (Fig. 4a). The top-ranking genes included *CACNA1H*, *MAFG*, *NOTCH1*, *GPSM1*, *MXRA8*, *PYCR1*, *VWA1* and *RXRA*. Of note, *CACNA1H* is a calcium channel gene previously implicated and experimentally validated to be important in ST-EPN-RELA cell survival [9], supporting the validity of our strategy in grading the significance of the genes. *NOTCH1* and *GPSM1* encode membrane proteins critical for Notch signaling and G-protein signaling, respectively, suggesting the involvement of these signaling pathways in ependymoma development in addition to NF-κB pathway that has received the most attention. To comprehensively nominate ependymoma-associated signaling pathways, we conducted a canonical pathway analysis with the genes that have more than one interacting chromatin site using Ingenuity Pathway Analysis (Qiagen) (Fig. 4b). Interestingly, the top enriched pathway was Notch signaling. Looking back at the ST-EPN-RELA gene list (Supplementary Table 2, online resource), 15 Notch signaling genes stood out, *ADAM12*, *CCND1*, *DLK1*, *DVL1*, *DVL2*, *HDAC1*, *HES1*, *HES5*, *JAG1*, *JAG2*, *LFNG*, *LNX1*, *MFAP2*, *NOTCH1* and *PSENEN*, further demonstrating Notch signaling might play a significant role in ST-EPN-RELA oncogenesis [2].

As we mentioned above, the fusion protein not only mediates promoter-promoter interactions but also enhancer-promoter interactions. To this end, we again made use of the 445 ST-EPN-RELA gene associated chromatin interactions identified in both G16-4 and BXD-1425-EPN cells, but focusing on the 91 non-promoter (putative enhancer) regions (Supplementary Table 4, online resource). We then counted the number of gene promoters connecting to these 91 enhancers. Although the majority of the enhancers had only one interacting promoter, some of them interacted with two to seven promoters (Supplementary Fig. 6a, online resource). The enhancer with the highest number of promoter interactions is located in the intron of *ASPSCR1* gene on chromosome 17, connecting with *ASPSCR1*, *DCXR*, *MAFG*, *MAFG-AS1*, *NPB*, *PYCR1* and *RAC3* genes (Supplementary Fig. 6b, online resource). More impressively, another distal enhancer had robust interactions with promoters of *NOTCH1* and *GPSM1* genes in both G16-4 and BXD-1425-EPN cells. Fusion protein binding was intensive and resulted in H3K27ac deposition at the enhancer region in G16-4 and BXD-1425-EPN cells (Fig. 4c). This suggests a potential regulatory scenario in which the fusion protein induces epigenetic changes at an enhancer and mediates chromatin reorganization with key genes of two distinct arms of signaling, specifically the Notch and G-protein signaling pathways, to initiate genetic programs underlying ST-EPN-RELA pathogenesis.

## Discussion

Informed by our study of epigenetic reprogramming driven by C11orf95-RELA fusion in HEK293T cell models and ependymoma cell line, we gained important knowledge in the molecular mechanisms underlying tumorigenesis driven by a single oncogenic C11orf95-RELA fusion [11, 14, 15]. Although the overexpression of C11orf95^fus1^ alone in HEK293T cells was not sufficient to drive ependymoma-like transcriptomic transformation, the ChIP-seq with both HEK293T and BXD-1425-EPN cells shows that the C11orf95 portion of the fusion plays a critical role binding to DNA and thus serving as a shuttle for the entry of RELA to the nucleus. By recognizing a specific DNA motif, C11orf95 portion targets particular genes and regulates their expression by hijacking the activation domain of RELA. Other than RELA, there was indication that C11orf95 also fused to other transcription factors such as YAP1 [12, 14] in ST-EPN-RELA, further implying the driving role of C11orf95. Since the first discovery of C11orf95-RELA mutation in ST-EPN, NF-κB pathway caught the most attention in tumorigenesis signaling [14, 15]. However, it is not the only pathway involved in ST-EPN-RELA [11]. Suggested by our chromatin interaction analysis in HEK293T and BXD-1425-EPN cell models, Notch signaling ranked among the top pathways involved in ST-EPN-RELA development, as a number of Notch genes were identified as chromatin hubs harboring more than one distal interacting partners. However, when we applied Notch inhibitor to treat BXD-1425-EPN cells, no significant effect on cell survival and growth was observed (data not shown). de Almeida Magalhães T et al. also had the same observation in their study [2]. They claimed although Notch inhibitor treatment did not change cell proliferation, apoptosis and colony formation of ST-EPN-RELA, it induced down-regulation of cancer stem cell markers. Therefore, we speculate on two possible scenarios regarding the roles of Notch signaling. The first possibility is that Notch signaling is not required for EPN cell maintenance, though it is essential for EPN development, as other signaling pathways may play substitutional roles such as the NF-κB and G-protein signaling pathways. Of note, NF-κB inhibitor alone did not change cell viability in ST-EPN-RELA, either [4], suggesting perhaps combinatorial treatment targeting multiple pathways could be pursued for ST-EPN-RELA therapeutics. Another possibility is that the efficacy of Notch inhibitor treatment on cell lines could be different from that of in vivo treatment in mice or human, since the microenvironment might be critical in determining the response of the cancer cells to treatment. The roles of the Notch, NF-κB, and G-protein pathways in EPN will warrant further in vivo investigations using single agent or combinations of inhibitors. Another interesting finding of this study is that other than the direct binding to gene promoters, C11orf95-RELA also bind to regulatory elements such as enhancers by chromatin interaction analysis. The enhancers identified to interact with multiple ST-EPN-RELA genes are potential targets for therapeutic interventions. Specifically, by disrupting one particular enhancer, more than one ST-EPN-RELA genes could be affected, providing a more efficient way to manipulate gene expression in ependymoma. While the present work presents the first epigenomic and transcriptomic characterization of the genetic program induced by the C11orf95-RELA fusion protein, future works on additional ST-EPN-RELA cell lines and primary tumors will further establish the core program as well as subtype differences critical for the development of precision and personalized therapeutics for this devastating cancer.

## Electronic supplementary material

Below is the link to the electronic supplementary material.

Supplementary file1 (DOCX 24244 kb)
