# Supplementary material for: C11orf95-RELA reprograms 3D epigenome in supratentorial ependymoma
Source: Acta Neuropathol. 2020 Sep 9;140(6):951–60. doi: 10.1007/s00401-020-02225-8 (PMC7666583; doi:10.1007/s00401-020-02225-8)
Supplement: Supplementary file 1 — Supplementary file1 (DOCX 24244 kb) [file 401_2020_2225_MOESM1_ESM.docx]

**C11orf95-RELA Reprograms 3D Epigenome in Supratentorial Ependymoma**

Jacqueline Jufen Zhu^1^, Nathaniel Jillette^1^, Xiao-Nan Li^2,3^, Albert Wu Cheng^1,4,5,6,*^, Ching C Lau^1,4,7,8,*^

^1^ The Jackson Laboratory for Genomic Medicine, Farmington, CT, USA

^2^ Texas Children's Cancer Center, Baylor College of Medicine, Houston, TX, USA

^3^ Department of Pediatrics, Northwestern University, Chicago, IL, USA

^4^ The Jackson Laboratory Cancer Center, Bar Harbor, ME, USA

^5^ Department of Genetics and Genome Sciences, University of Connecticut Health Center, Farmington, CT, USA

^6^ Institute for Systems Genomics, University of Connecticut Health Center, Farmington, CT, USA

^7^ Division of Hematology-Oncology, Connecticut Children’s Medical Center, Hartford, CT, USA

^8^ Department of Pediatrics, University of Connecticut Health Center, Farmington, CT, USA

^*^ Corresponding authors ([albert.cheng@jax.org](mailto:albert.cheng@jax.org), [ching.lau@jax.org](mailto:ching.lau@jax.org))

**Supplementary material**

**
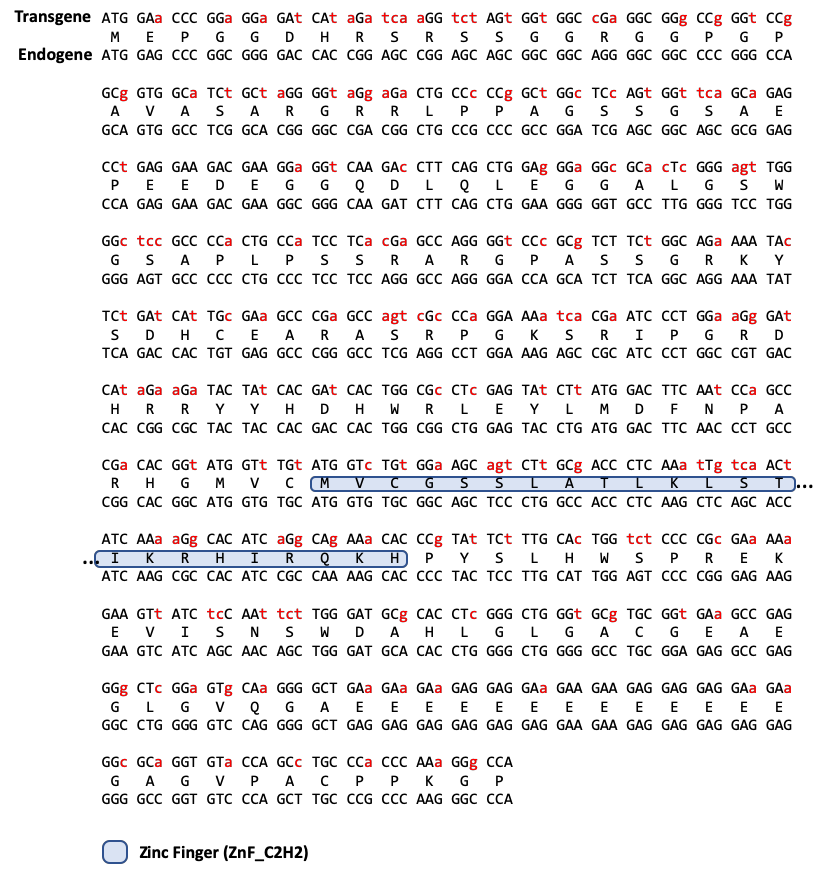
**

**Supplementary Figure 1**

DNA and protein sequence of C11or95fus1. Upper and lower DNA sequence tracks show open reading frames of C11orf95fus1 transgene and endogenous gene, respectively. Red lowercase nucleotides denote synonymous changes made to the transgenic DNA sequence to allow differentiation from endogenous DNA sequence. Middle amino acid track shows the protein sequence. A predicted C2H2-type zinc finger is highlighted in the protein sequence track.

**
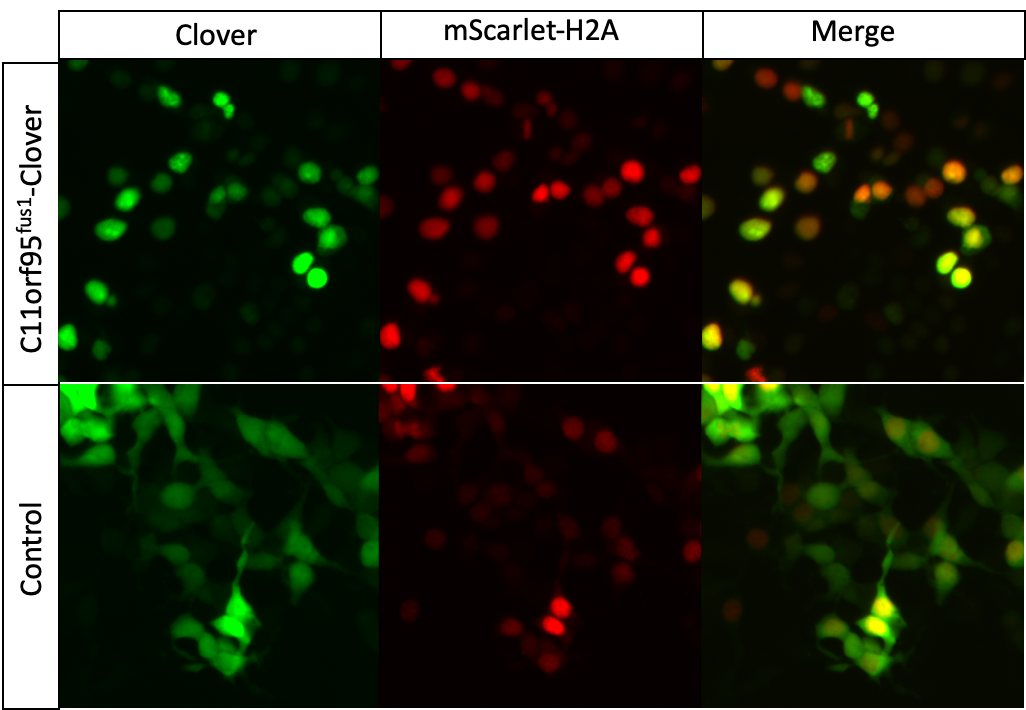
**

**Supplementary Figure 2**

Colocalization of C11orf95^fus1^ with cell nucleus. Clover indicate C11orf95^fus1^ fused or unfused clover protein in C11orf95^fus1^-clover or control cells, respectively. mScarlet-H2A indicate cell nucleus.

**
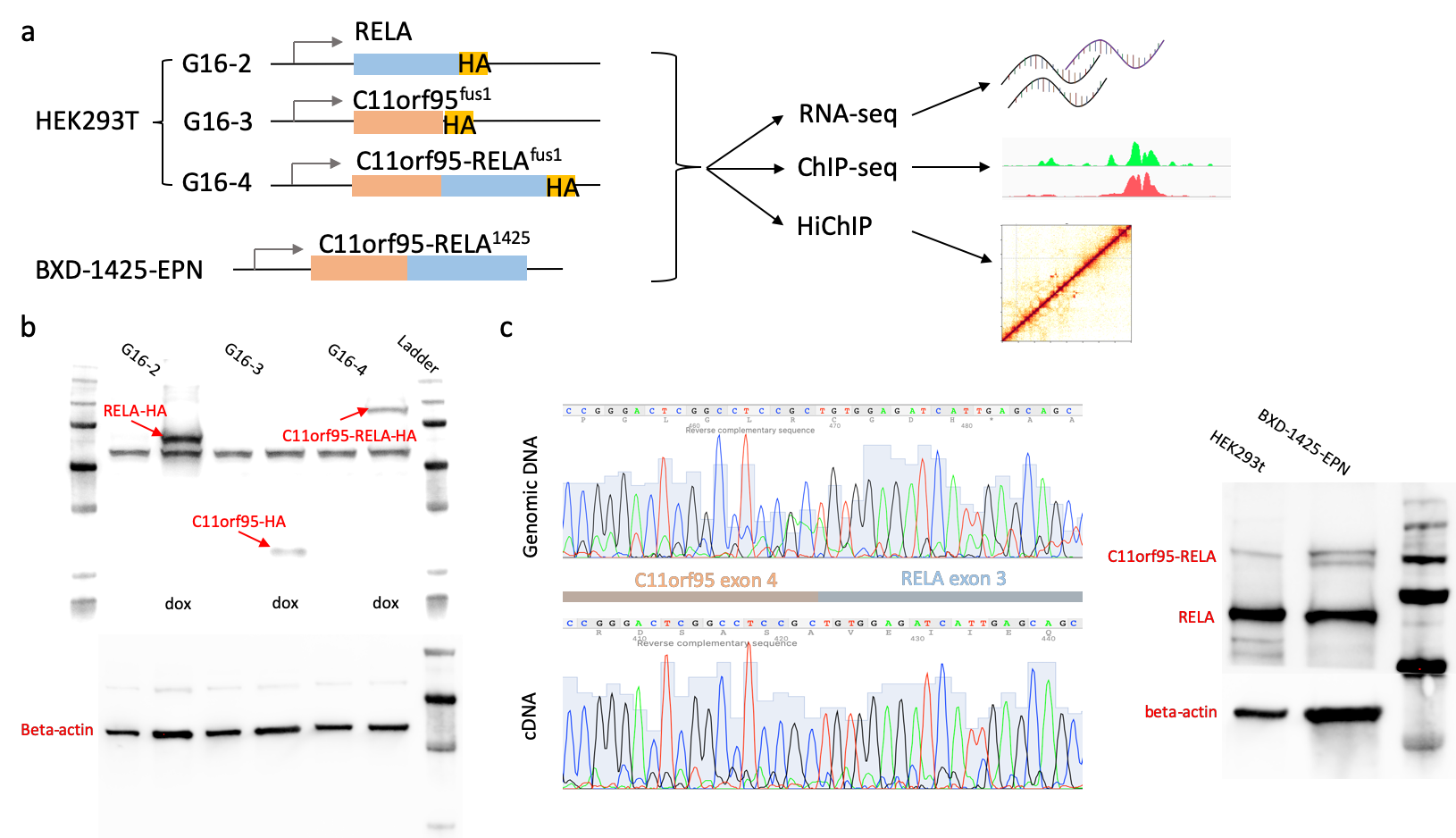
**

**Supplementary Figure 3**

a. Flow chart of different cell lines used for different sequencing mappings.

b. Western blot for RELA, C11orf95fus1 and C11orf95-RELAfus1 overexpression in G16-2, G16-3, G16-4 cells. Red arrows point to the overexpressed proteins in each cell line. Each cell line has two lanes. One lane is for doxycycline-treated cells, the other is for non-treated cells as control. Beta-actin is used as internal protein control for each cell type.

c. Confirmation of fusion type in BXD-1425-EPN cells with genotyping and western blot.

**
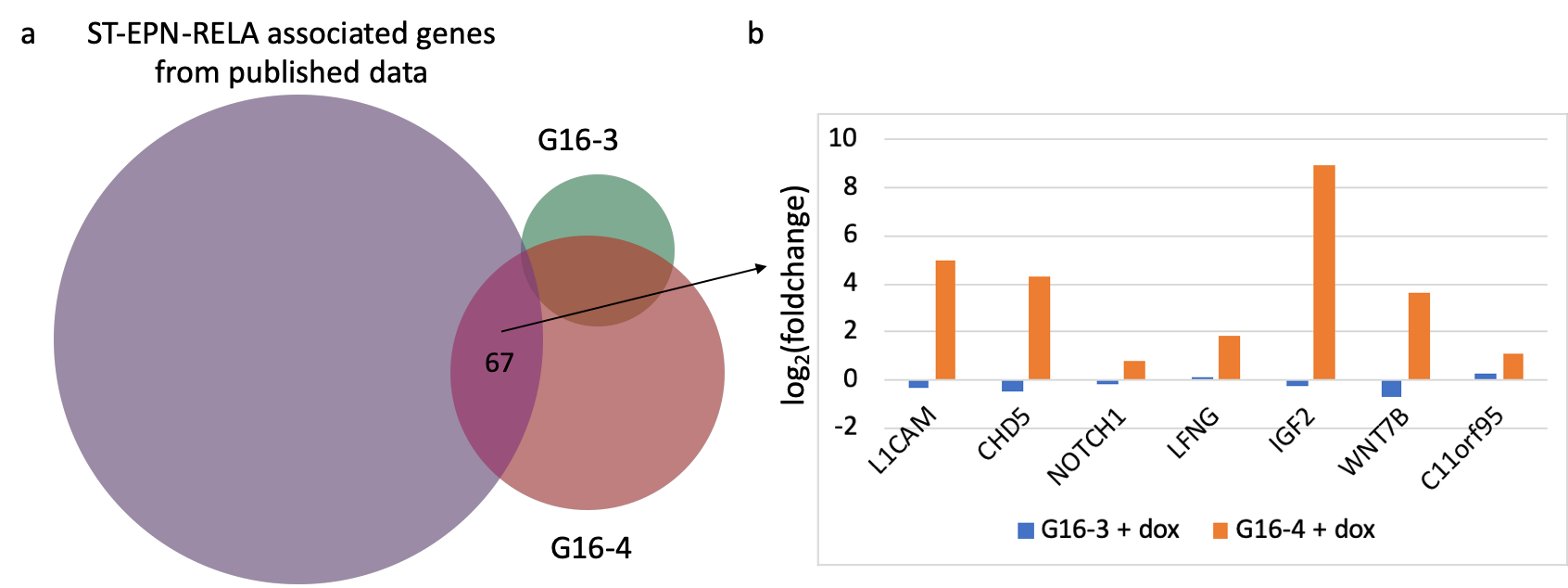
**

**Supplementary Figure 4**

a. Venn diagram of ST-EPN-RELA associated genes from published data (supplementary Table 2) (purple) and upregulated genes in G16-3 (green) and G16-4 cells (red). Upregulated genes in G16-3 and G16-4 cells were identified by RNA-seq by comparing to HEK293T cells. There were very few overlaps of G16-3 upregulated genes and published ST-EPN-RELA genes, but 67 genes were upregulated in G16-4 cells including the well-known ST-EPN-RELA associated genes (*L1CAM*, *CHD5*, *NOTCH1*, *LFNG*, *IGF2*, *WNT7B* and *C11orf95*). The Venn diagram was not drawn to scale.

b. Expression of well-known ST-EPN-RELA associated genes in G16-3 and G16-4 cells.

**
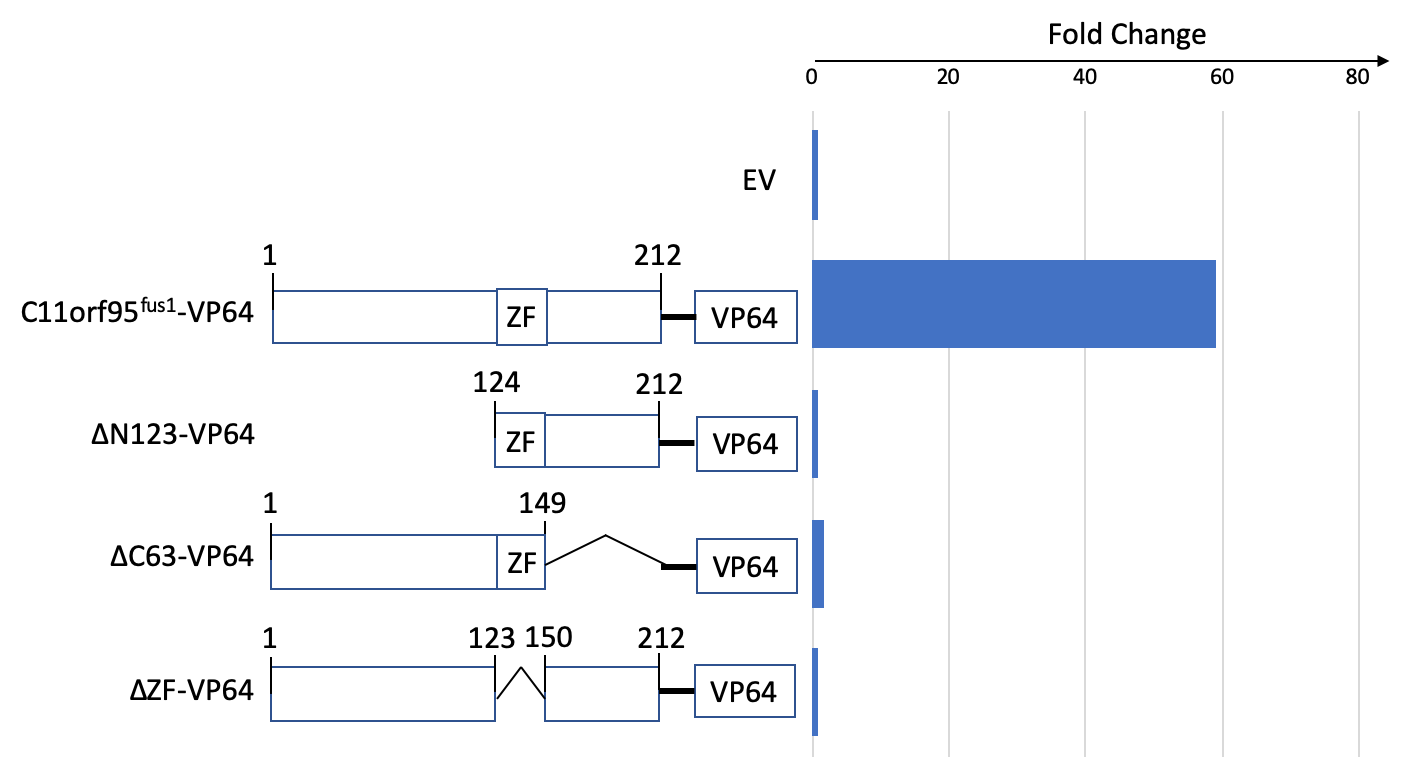
**

**Supplementary Figure 5**

Truncation analysis of C11orf95^fus1^-VP64. Three truncation mutants of C11orf95^fus1^-VP64 were generated by deleting 123 amino acids (a.a.) from the N-terminus (ΔN123-VP64), deleting 63 a.a. from the C-terminus (ΔC63-VP64), or deleting the zinc finger domain (ZF) (a.a. 124-149, ΔZF-VP64) of the C11orf95^fus1^ fragment. Transactivation activities of the three mutants were assayed by the EGFP reporter with 15xC11orf95 DNA binding motifs. Empty vector (EV) and the fold changes in mean GFP fluorescence relative to the EV control (set to 1) are displayed in the bar chart.

**
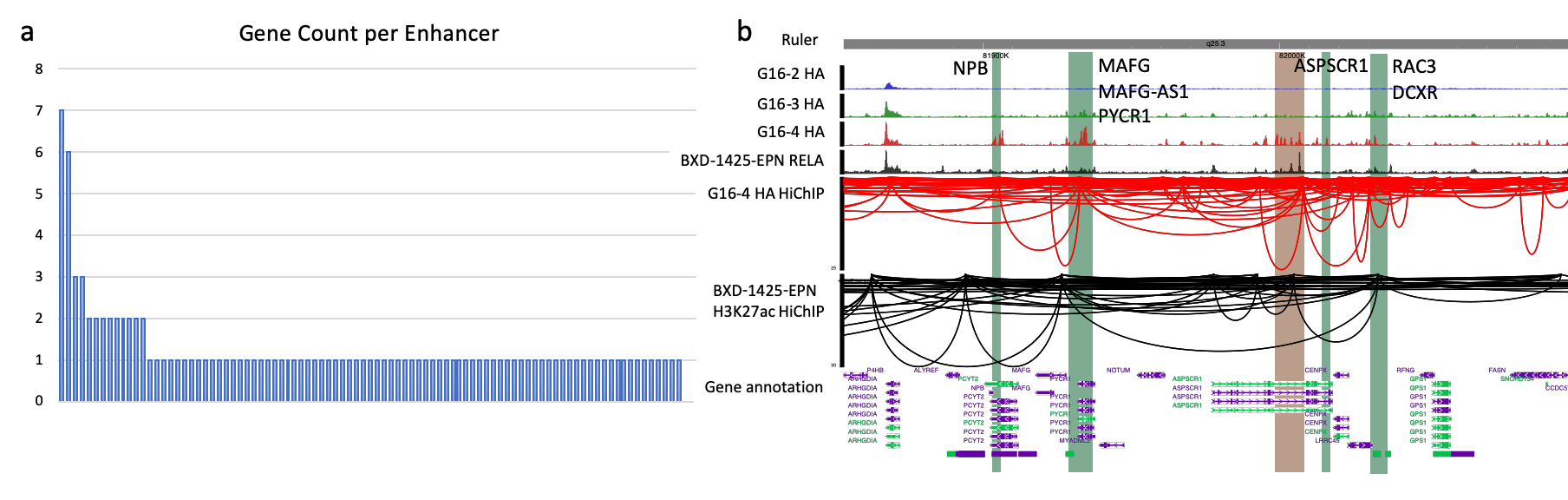
**

**Supplementary Figure 6**

a. Histogram of interaction counts per enhancer. 115 ST-EPN-RELA associated enhancer-promoter interactions were analyzed. The counts indicate the number of ST-EPN-RELA genes that interact with the same enhancer.

b. Browser view of the enhancer that targets seven ST-EPN-RELA genes. The green bars indicate gene promoters. The orange bar indicates the enhancer.

**Supplementary Table 1 Gene ontology enrichment of ChIP-seq identified genes**

| C11orf95fus1 C11orf95-RELA fus1 common binding genes | | | | | | |
| --- | --- | --- | --- | --- | --- | --- |
| Category | Term | Description | Log(p-value) | Log(q-value) | InTerm_InList | Gene symbols |
| GO Biological Processes | GO:0007050 | cell cycle arrest | -8.6111501 | -4.292 | 29/236 | ABL1,BIN1,ZFHX3,CCND1,CDK6,CDKN2B,ARID3A,INHA,IRF1,NBN,PLAGL1,PRKAB2,PRKACA,SETMAR,TCF7L2,TFAP4,TGFB1,TP73,MAGI2,GAS2L1,ZNF268,LAMTOR2,TRIAP1,RRAGD,MLST8,PPP1R9B,TP53INP1,MIR200B,KLLN,BCL2L1,BMP7,SEPTIN7,MAPK14,DNA2,HHEX,NR4A1,NUBP1,NPR2,PTEN,RRM2,TAF6,PSMF1,MAD2L1BP,TEX14,CTDSP1,ZNF655,FBXO31,JADE1,DOT1L,FGF8,NUMA1,PTCH1,MAP4K2,MAP2K4,TUBA1A,ZMPSTE24,PIM2,CEP43,SIRT2,USP22,OBSL1,ANKRD17,STK39,MTA3,CCSAP,RHOA,CSNK2A1,MSX1,BIRC6,FAM83D |
| GO Biological Processes | GO:0010506 | regulation of autophagy | -7.8448139 | -3.827 | 34/334 | ABL1,BOK,CAPNS1,EEF1A2,FOXO1,GAPDH,GOLGA2,PRKAB2,PRKACA,VDAC1,USP10,DEPDC5,ATG13,BCL2L11,DNM1L,ZMPSTE24,PIM2,CDC37,SIRT2,ATP13A2,GPSM1,LAMP3,LAMTOR2,VPS13C,VPS13D,UBQLN4,TIGAR,RRAGD,MLST8,TRIM8,RAB1B,LRSAM1,TP53INP1,TRIM65,CSNK2A1,SQSTM1,HGS,RGS19,PACS2,MTERF3,ABCD1,CBFA2T3,DVL1,PTEN,ZFP36,BCAP31,TOB1,DISC1,TRIB2,PACSIN3,PRR5L,ITCH |
| GO Biological Processes | GO:0010821 | regulation of mitochondrion organization | -6.8442188 | -3.148 | 23/190 | BCL2L1,BCL2L2,BOK,OPA1,PLAUR,TP73,VDAC1,YWHAB,BAP1,SQSTM1,PDCD5,ATG13,BCL2L11,DNM1L,CDC37,ATP13A2,SIRT4,TRIAP1,VPS13C,VPS13D,MFF,TIGAR,MICALL2,ATP2A1,PIM2,RTL10,MSX1,BCAP31,TXNDC12,MARCHF7,ELL3,DNA2,EPAS1,ESRRA,NDUFAF6,SLC25A10,PRKAB2,MPC1 |
|  | | | | | | |
| C11orf95fus1 unique binding genes | | | | | | |
| Category | Term | Description | Log(p-value) | Log(q-value) | InTerm_InList | Gene symbols |
| GO Biological Processes | GO:0006412 | translation | -6.7922508 | -2.473 | 61/723 | APP,CIRBP,DHX9,EEF2,EIF4A1,EIF4EBP1,GSPT1,HNRNPL,MRPL58,IGHMBP2,EIF6,MTIF2,NCBP1,YBX1,MAPK3,PTBP1,RPL21,RPL36AL,RPL41,RPS2,RPS8,RRBP1,ATXN2,SHMT2,SRP9,TARBP2,AIMP2,CNOT8,EIF4E2,MATR3,EIF1,RIDA,EIF1B,RACK1,MRPL28,KHDRBS1,GCN1,RNF139,CNOT1,PPP1R15A,RPL36,ZNF385A,MRPL46,PABPC1,MRPL15,EEF2K,DPH5,CNOT11,EIF4ENIF1,MIF4GD,MRPS11,CARS2,AARSD1,SESN2,EARS2,MRPL55,CPEB2,LSM14B,ZNF540,MRPL21,EIF2AK4,SORL1 |
| GO Biological Processes | GO:1901137 | carbohydrate derivative biosynthetic process | -6.1177909 | -2.276 | 62/774 | ACLY,ADA,ADCY3,ADCY5,ADK,ADSL,ADSS2,APP,DUT,FUT8,GALK1,GCNT1,HYAL1,EIF6,LMAN1,MAN2A1,MGAT2,MPI,PGM3,RENBP,RPN2,ST6GAL1,SLC2A4,SLC25A1,SOAT1,MOGS,CHST1,GMPS,ST3GAL5,B4GALT5,PIGL,NCOR1,GFPT2,PIGK,CSPG5,MPC2,GLCE,HSD17B12,CMPK1,PANK1,CHST12,POGLUT1,MAN1C1,GALNT16,ELOVL5,GOLPH3,LMF1,CHPF,ALG13,PGAP1,PIGZ,ST6GALNAC5,SEH1L,B3GNT5,ACSS1,GAL3ST3,MCFD2,NUS1,CANT1,TYW5,ACSF3,CHSY3,RNF139,ERP44,MAN2B2,CLN5 |
| Reactome Gene Sets | R-HSA-446203 | Asparagine N-linked glycosylation | -5.778288 | -2.061 | 32/304 | ARF4,CANX,FUT8,LMAN1,MAN2A1,MGAT2,MPI,PGM3,RENBP,RPN2,ST6GAL1,SPTB,STX5,MOGS,ST3GAL5,B4GALT5,SEC24D,SEC16A,GFPT2,YKT6,RNF139,SEC31A,TMED3,SLC17A5,SAR1B,STX17,MAN1C1,ALG13,ST6GALNAC5,TRAPPC9,MCFD2,NUS1,CENPE,HTT,SORL1,SURF4,PLPP3,CYTH3,SCAMP2,RACK1,TRAPPC8,RACGAP1,NRBP1,EXOC1,GOLPH3,LMF1,PGAP1,LRRK2,DNM1,SLC2A4,SNAP91,WDR11 |
|  | | | | | | |
| C11orf95-RELAfus1 unique binding genes | | | | | | |
| Category | Term | Description | Log(p-value) | Log(q-value) | InTerm_InList | Gene symbols |
| GO Biological Processes | GO:0048812 | neuron projection morphogenesis | -10.146082 | -5.958 | 77/658 | ADORA2A,ARHGAP4,BMPR1B,BSG,CRABP2,CREB1,DBN1,EFNA2,CELSR3,EPHB2,ERBB2,FYN,GFRA3,GLI2,GRB7,MNX1,L1CAM,SMAD4,MAP1B,MEF2A,MOV10,NBL1,NEO1,NFATC4,NPTX1,NTF4,NTRK2,PAFAH1B1,NECTIN1,RAC2,CXCL12,SKIL,SLIT1,SPTBN2,SRC,STK4,VLDLR,WNT7A,WNT7B,ULK1,CNTNAP1,CPNE1,BRSK2,B4GALT6,B4GALT5,LHX2,CUL7,UST,PLXNC1,BAIAP2,SEMA4D,VAX1,PLXNB2,TSKU,NSMF,SIPA1L1,SS18L1,SH3KBP1,BCL11A,ROBO4,DSCAML1,LGR6,SEMA4A,RTN4R,PREX2,NDEL1,NTNG2,KNDC1,WNT3A,UNC5A,BOC,ARHGAP33,NRN1L,VSTM2L,DAB2IP,PHACTR1,AGRN,PACSIN2,ACTN4,AXL,CASP9,HES1,LAMA5,MYH9,MYO7A,NBEAL2,CLIC4,LIMS2,RCC2,COL20A1,VWA1,COL18A1,FERMT3,TBC1D20,CCDC88C,CTNNA1,STK24,MAPK8IP3,CRTAC1,CTSZ,KIT,LYN,NFE2L2,ROR2,P2RX4,PALM,PFN1,PODXL,PTK6,TRPM2,WAS,FZD1,CBFA2T2,NEURL1,IFT140,HDAC4,TBC1D5,SEPTIN9,TBC1D8,RABGAP1,TBC1D22A,TBC1D10B,WRAP73,TBC1D14,TRPV4,ITM2C,ESPN,ACAP3,ARAP1,TBC1D16,PLK5,PROM2,TBC1D10C,ITGA7,ITGB2,MKLN1,S100A13,ZMYM5,RHOBTB1,RHOF,STRIP1,FMNL3,SH3D19,SYNE3,BCL9L,BCL6,DLX2,MYCL,REST,SOX3,TCF3,ZNF536,OLIG2,HEYL,GPRC5B,CASZ1,DUOXA1,ADGRB2,HDAC1,OXT,GJC2,SYNDIG1,NKX6-2,GSX2,DUSP1,S1PR1,CCR10,CXCL2,HOXB9,IL12A,MST1,PDE4B,PDGFA,PTPRJ,SAA1,BCAR1,ADGRE2,CYRIB,MIR424,PIK3R2,RGS3,PIK3R3,LRRC4,CLDN5 |
| GO Biological Processes | GO:0030029 | actin filament-based process | -6.4552824 | -3.178 | 76/785 | ABR,ACTN4,AQP1,BCL6,BCR,CACNA1C,CSF3,DBN1,S1PR1,KCNJ3,KCNJ5,KIT,KRT19,LLGL2,MARCKS,SMAD4,MEF2A,MKLN1,MYH7,MYH9,MYL3,MYO7A,PAFAH1B1,PDE4B,PDGFA,PFN1,PRKAR1A,RAC2,CXCL12,SPTBN2,SRC,TNNI2,TNNT3,TRPM2,WAS,BRSK2,HIP1R,TRIP10,PICK1,BCAR1,CELSR1,RHOBTB1,IQSEC1,BAIAP2,PACSIN2,IQSEC2,SH3BP1,FSCN2,SIPA1L1,ARFIP1,PHPT1,SH3KBP1,VILL,AMOTL2,SNX9,CYRIB,RHOF,CDC42BPG,TRPV4,MYO1G,ESPN,OBSCN,STRIP1,FMNL3,ARAP1,ARHGEF19,PPARGC1B,CLDN19,AMOTL1,PHACTR1,KLHL17,SMTNL2,RNF207,MIR133A2,CCDC88C,SMIM22,AEBP1,ARL2,CDKN1B,COMP,FOXC1,KRT17,MAP1B,EMILIN1,EFEMP2,NDEL1,EPPK1,ADAMTS14,KIF18B,FIGNL2 |
| GO Biological Processes | GO:0048705 | skeletal system morphogenesis | -6.2403309 | -3.000 | 33/240 | ANXA6,BMPR1B,COMP,DLX2,FOXC1,HOXB3,HOXB4,HOXB5,HOXB6,HOXB9,HOXD8,HYAL1,LRP5,LTBP3,LTF,ROR2,PKD1,POR,TBX1,WNT7A,WNT9A,IFT140,SATB2,CYP26B1,DSCAML1,WDR48,COL20A1,TRPV4,VWA1,COL27A1,LRP5L,PPARGC1B,UNCX,BGN,GLI2,GPLD1,HOXA10,HOXC10,IGF2,JAG2,KIT,PAFAH1B1,PTH1R,SRC,NSD2,WNT7B,HDAC4,SH2B3,SEMA4D,RAI1,SLC39A14,FOXP1,FGFRL1,LRRK1,WFIKKN2,SPNS2,PDGFA,RORC,UMODL1,SLC25A25 |

**Supplementary Table 2 ST-EPN-RELA genes from publications**

| 886 ST-EPN-RELA genes |
| --- |
| ABCA8,ABCB1,ABCB4,ABCG1,ABCG2,ABL1,ABLIM3,ABTB2,ACOT7,ACP2,ACPP,ACSS1,ACTN2,ACVRL1,ADAM12,ADAM33,ADAMDEC1,ADAMTS19,ADAMTS2,ADAMTSL2,ADAMTSL4,ADAP1,ADAR,ADORA2B,AFAP1L1,AFAP1L2,AIF1L,AIM2,AIMP2,AKIRIN2,AKT3,ALAS1,ALDH1A3,ALDH3A1,ALDH4A1,ALMS1,ALS2CL,AMD1,ANGPT2,ANGPTL6,ANKDD1A,ANKRD13B,ANKRD65,ANKRD9,AP5B1,APBA2,APMAP,ARAP1,ARAP3,ARC,AREL1,ARHGAP11B,ARHGAP19-SLIT1,ARHGAP28,ARHGAP4,ARHGEF3,ARID3B,ARID4B,ARL16,ASCL2,ASPM,ASPSCR1,ATAD3C,ATG4A,ATP10D,ATP11A,ATP6V1C2,ATXN1L,AVL9,AXIN2,AXL,B3GALT1,B4GALT6,B4GALT7,BAI1,BAIAP2,BCL11A,BCL2,BCL2L1,BCL3,BCOR,BEGAIN,BEST3,BIK,BIRC3,BIRC5,BLM,BMP2,BMP2K,BMPER,BOK,BOP1,BORA,BRINP2,BRSK2,BSN,BTBD11,BTBD6,BUB1,C11ORF84,C11ORF95,C12ORF5,C17ORF62,C1ORF61,C20ORF195,C2CD4C,C5ORF34,CA8,CABP7,CACNA1B,CACNA1G,CACNA1H,CACNA1I,CACNA2D2,CADM3,CALB1,CAMK2B,CAMK2N1,CARD16,CARHSP1,CASKIN2,CASP1,CASP4,CASZ1,CBFA2T3,CBLN2,CBX2,CBX4,CCDC24,CCL2,CCL3,CCND1,CCND3,CCSER1,CD34,CD40,CD44,CD55,CD82,CD93,CDC25A,CDC7,CDCA3,CDH10,CDH22,CDK1,CDK2,CDKN1C,CEBPG,CELF3,CELSR3,CEP68,CERK,CETP,CHAF1B,CHD5,CHRDL2,CHST1,CHST12,CHSY1,CHSY3,CISH,CKAP2L,CLDN10,CLDN5,CMIP,CMTM8,CMTR2,CNTNAP2,COBLL1,COL18A1,COL1A1,COL1A2,COL3A1,COL4A1,COL4A2,COL5A2,COL6A1,COL9A3,COMP,CORIN,COX19,CPEB1,CPT1C,CRABP2,CREG1,CRH,CROCC,CROT,CRYBA4,CRYM,CSF1,CSNK1G1,CSPG4,CSPG5,CTSK,CUX2,CX3CL1,CXCL1,CXCL10,CXCL2,CXCL6,CYB561D2,CYP27C1,CYYR1,DACH1,DACT1,DCN,DCX,DCXR,DDX25,DDX49,DDX52,DEPDC1B,DGAT1,DHH,DHX58,DIO2,DIO3OS,DISC1,DISP2,DKK1,DKK2,DLEU2,DLG5,DLGAP5,DLK1,DMRT2,DNAH14,DNAJC12,DNM1L,DOCK3,DOK4,DPP9,DPYSL4,DPYSL5,DRAXIN,DSP,DTL,DUSP2,DUSP6,DVL1,DVL2,EBF2,ECEL1,ECSCR,ECT2,EDA,EEF1A2,EFCAB4A,EFHD2,EFNA2,EGFL6,EHD3,EIF4G1,ELAVL3,ELF3,ELF4,ELFN1,ELL3,ELN,ELTD1,EME2,EML4,ENPEP,ENPP1,EOGT,EOMES,EPHA2,EPHB2,EPHB3,EPHB4,EPPK1,ERG,ESAM,ESPL1,ESX1,ETS1,ETV3,EVL,EXOC7,FAM101B,FAM135A,FAM155A,FAM155B,FAM167A,FAM184B,FAM211A,FAM212B,FAM216A,FAM43A,FAM83D,FAM83G,FAM84A,FANCI,FANCM,FAR2,FAT4,FBLL1,FBLN2,FBP2,FBXO31,FGF18,FGFRL1,FHDC1,FLRT3,FLT1,FMNL3,FMOD,FN1,FOXF1,FOXF2,FOXM1,FOXN4,FOXO6,FREM2,FRK,FRMD4A,FRZB,FTH1,FZD1,FZD10,FZD2,FZD6,FZD7,FZD9,GABRA3,GABRG3,GAD1,GALE,GAREML,GAS2L3,GATA5,GCGR,GCH1,GCNT2,GDF10,GEN1,GLI2,GLI3,GLIS3,GNAL,GNG3,GPC1,GPC3,GPR116,GPR124,GPR137C,GPR153,GPR161,GPR85,GPR98,GPSM1,GPX3,GRB10,GRB14,GRK5,GRM3,GSE1,GTF2IRD1,GTSE1,GUCY1B2,HAPLN1,HDAC1,HDAC4,HELLS,HERC6,HES1,HES3,HES4,HES5,HIGD1B,HJURP,HMCN1,HMHA1,HMX1,HOGA1,HOMER2,HS3ST3A1,HS3ST3B1,HSPB1,HSPG2,IBA57,ICAM1,ICMT,IDE,IER2,IFI30,IFNGR2,IGDCC3,IGF2,IGF2-AS,IGSF9,IKBIP,IL13RA1,IL15RA,IL18R1,IL1R1,IL1RAPL1,IL1RN,IL20RA,IL4RA,IL6,ILVBL,IMPDH1,ING5,INHBB,INPP4B,INPP5A,INSR,INTS1,INTS7,IQGAP2,IQSEC1,IRF1,IRF2,IRF7,ISOC1,ITGA1,ITGA10,ITGB1,ITM2A,ITPKC,JADE3,JAG1,JAG2,KBTBD12,KBTBD2,KCNA3,KCNE3,KCNH6,KCNJ4,KCNK10,KCNK3,KCNK6,KCNQ2,KCNT2,KDM4A,KDM4B,KIAA0101,KIAA1244,KIF20A,KIF21B,KIF23,KIF26A,KIF26B,KIF7,KIFC2,KLF12,KLHDC5,KLHL25,KLHL42,KRI1,KRT75,L1CAM,L3MBTL1,LAMA5,LAMP3,LARP4,LBX1-AS1,LEF1,LEPRE1,LFNG,LHX2,LIF,LIN7A,LINC00263,LINC00606,LINC00982,LINGO1,LITAF,LMO1,LNPEP,LNX1,LPAR3,LPCAT1,LPHN2,LPL,LRP4,LRP8,LRPAP1,LRRC17,LRRC3DN,LRRTM4,LSP1,LYN,LYPLA2,LYST,MAEA,MAFG,MAFG-AS1,MAGEL2,MAGI3,MALT1,MAMDC4,MANEAL,MAP2K3,MAP3K3,MAP4K2,MAPK8IP1,MAPKAPK3,MBD6,MCAM,MCF2L,MCM10,MCM3AP-AS1,MCMBP,MDM2,MECOM,MED30,MEG3,MEGF6,MERTK,MEST,METRNL,MEX3A,MEX3B,MFAP2,MFGE8,MFSD11,MGLL,MICALL2,MIF,MIR145,MKNK2,MLKL,MMP16,MPZL2,MRGBP,MSANTD2,MSI1,MSI2,MSX1,MTG1,MTSS1,MTSS1L,MUC12,MXD3,MXRA5,MXRA8,MYH9,MYLIP,MYO1B,MYO3A,N4BP3,NAA40,NAT1,NAV1,NCAN,NCAPG,NCAPH,NCOA7,NDC80,NES,NFAT5,NFATC4,NFKB1,NFKB2,NFKBIA,NHS,NID1,NMU,NOL9,NOTCH1,NOTCH2NL,NOTUM,NOX4,NPB,NPY1R,NRARP,NRIP1,NRIP2,NRP1,NRXN1,NRXN2,NUDT17,NUP210,NUPL1,NUSAP1,NXN,OAF,OBSL1,ONECUT2,OSBP2,P2RX5,PABPC4L,PAG1,PAK7,PANK1,PAPSS2,PARP12,PARP14,PARP4,PARP9,PBK,PBX3,PCDH12,PCDH20,PCDH8,PCLO,PCOLCE,PCP4L1,PDCD5,PDE10A,PDGFA,PDGFB,PDGFRB,PDZD2,PDZRN4,PEX11A,PGAP1,PGF,PHF23,PHIP,PHLDA1,PHLDA2,PIDD,PIK3R2,PIK3R3,PIM3,PIP5K1B,PKDCC,PLA2G7,PLCH2,PLEKHG2,PLEKHG4B,PLG,PLIN2,PLXDC1,PLXNB2,PMAIP1,PMEPA1,PNOC,POLR2C,POMGNT1,POU2F1,PPFIA3,PPP4R1,PQLC2,PRDM2,PRKAB2,PRKCD,PROM1,PRR5,PRSS12,PRSS35,PSD2,PSENEN,PSME1,PTGS2,PTH1R,PTHLH,PTN,PTP4A3,PTPRG,PTPRS,PWWP2B,PYCR1,QPCT,QSOX1,RAB11FIP4,RAB26,RAB33A,RAB34,RAB3IP,RAB7B,RAC3,RACGAP1,RAD54B,RAI2,RALYL,RAP1GAP,RASEF,RASIP1,RBPMS2,RCOR2,RDH10,RELA,RELB,RERG,RFPL1S,RMND5A,RNF103,RNF144B,RNF43,ROBO1,ROBO4,ROR2,RORB,RPRM,RRAS2,RRM2,RSPO4,RTN4R,RUNDC3A,RUNDC3B,RXRA,S100A2,S100A6,SAMD14,SAPCD2,SCARA3,SCARF1,SCG3,SCML1,SCML2,SCN3A,SCN8A,SDC1,SDC4,SDHAP3,SEL1L3,SEMA6A,SEZ6L,SH2D5,SH3BP2,SH3BP5,SHB,SHC1,SLC12A7,SLC27A3,SLC29A4,SLC2A1,SLC2A4RG,SLC2A6,SLC35E4,SLC37A1,SLC38A5,SLC46A1,SLC4A11,SLC6A11,SLC6A6,SLC7A10,SLC7A6,SMAD7,SMC4,SMIM3,SMO,SNAI2,SNORD113-3,SNORD114-3,SOGA2,SOGA3,SORCS2,SOWAHC,SP5,SPATA6,SPCS3,SPINT2,SPRY1,SRPK3,SSBP3,SSTR5-AS1,ST6GALNAC3,ST8SIA2,ST8SIA3,STARD10,STARD4,STAT5A,STC1,STMN4,STOX2,STRA13,STRADA,SUZ12,SYNDIG1,SYNGR1,SYT7,Sep-09,TAF5,TAF7L,TAL2,TAP1,TAPBP,TBC1D16,TBC1D9,TBX1,TBX3,TBX5,TBX5-AS1,TCERG1L,TCF3,TCIRG1,TDO2,TFPI,TFPI2,TGIF2,TGM2,TGS1,THBS1,TIMELESS,TLE1,TLX1,TM7SF2,TMC6,TMEM132C,TMEM2,TMEM200B,TMEM200C,TMEM255B,TMEM35,TMEM45B,TMEM51,TMEM57,TMEM63C,TMEM74B,TMTC4,TNFAIP3,TNFAIP8L1,TNFRSF18,TNFRSF4,TNIP1,TNIP2,TNRC6C-AS1,TOP2A,TOX3,TP73,TPBG,TPSG1,TRAM1L1,TRAM2,TRIB2,TRIP6,TRPC6,TSHR,TSNARE1,TSPAN15,TSPAN33,TSPAN4,TTC9,TXNRD1,TYMS,UBAP2L,UBE2C,UBE2QL1,UNC119,UNC5A,UNC5B,USP24,VASH1,VASN,VAV2,VIM,VIPR1,VPS9D1,VSIG10L,VSNL1,VWA1,WDR82,WFIKKN2,WIF1,WNT11,WNT2B,WNT7B,WRAP73,WSCD1,YAE1D1,ZC3H8,ZDHHC22,ZFHX4,ZFHX4-AS1,ZFPM2,ZIC1,ZIC2,ZIC5,ZMIZ1,ZMYM5,ZNF133,ZNF14,ZNF185,ZNF195,ZNF300,ZNF362,ZNF385C,ZNF491,ZNF514,ZNF518B,ZNF521,ZNF536,ZNF589,ZNF655,ZNF704,ZNF75A,ZNF786,ZNRF3,ZWINT, |

**Supplementary Table 3 Chromatin interaction counts of identified ST-EPN-RELA genes**

| Gene symbol | Interaction counts |
| --- | --- |
| MAFG | 10 |
| CACNA1H | 10 |
| PYCR1 | 9 |
| NOTCH1 | 9 |
| MXRA8 | 9 |
| MAFG-AS1 | 9 |
| GPSM1 | 9 |
| VWA1 | 8 |
| RXRA | 8 |
| PLXNB2 | 7 |
| MEGF6 | 7 |
| LAMA5 | 7 |
| TBX1 | 6 |
| PLCH2 | 6 |
| PIM3 | 6 |
| NPB | 6 |
| LINC00982 | 6 |
| GATA5 | 6 |
| SH3BP2 | 5 |
| SAPCD2 | 5 |
| RAC3 | 5 |
| LFNG | 5 |
| KIFC2 | 5 |
| IGF2 | 5 |
| HES4 | 5 |
| FBLN2 | 5 |
| EFNA2 | 5 |
| COX19 | 5 |
| SLC6A6 | 4 |
| RCOR2 | 4 |
| RAB26 | 4 |
| MXD3 | 4 |
| MRGBP | 4 |
| MAP4K2 | 4 |
| MAEA | 4 |
| ING5 | 4 |
| EPPK1 | 4 |
| CRABP2 | 4 |
| CBX4 | 4 |
| BOP1 | 4 |
| ASPSCR1 | 4 |
| ARL16 | 4 |
| TCIRG1 | 3 |
| RRM2 | 3 |
| RAB34 | 3 |
| NES | 3 |
| MAPKAPK3 | 3 |
| MAP2K3 | 3 |
| KLHL25 | 3 |
| KIF26A | 3 |
| JAG2 | 3 |
| INPP5A | 3 |
| IGF2-AS | 3 |
| IBA57 | 3 |
| GCGR | 3 |
| DGAT1 | 3 |
| DCXR | 3 |
| CROCC | 3 |
| COL9A3 | 3 |
| CMIP | 3 |
| CLDN5 | 3 |
| CISH | 3 |
| AP5B1 | 3 |
| ADAP1 | 3 |
| ACOT7 | 3 |
| ZNRF3 | 2 |
| TRIP6 | 2 |
| TNIP1 | 2 |
| TMEM63C | 2 |
| TMC6 | 2 |
| TLX1 | 2 |
| STAT5A | 2 |
| SSTR5-AS1 | 2 |
| SLC35E4 | 2 |
| SLC2A4RG | 2 |
| SH2D5 | 2 |
| S100A6 | 2 |
| POLR2C | 2 |
| PLEKHG2 | 2 |
| OSBP2 | 2 |
| NRARP | 2 |
| MAMDC4 | 2 |
| LBX1-AS1 | 2 |
| KRI1 | 2 |
| KDM4B | 2 |
| IER2 | 2 |
| HOGA1 | 2 |
| FMNL3 | 2 |
| FGFRL1 | 2 |
| EPHB4 | 2 |
| ELFN1 | 2 |
| DVL1 | 2 |
| DOK4 | 2 |
| CYB561D2 | 2 |
| CHSY1 | 2 |
| CHD5 | 2 |
| CELSR3 | 2 |
| CBX2 | 2 |
| CASZ1 | 2 |
| CASKIN2 | 2 |
| ANKRD65 | 2 |
| ADAMTSL4 | 2 |
| ZNF786 | 1 |
| ZNF589 | 1 |
| VAV2 | 1 |
| UNC5B | 1 |
| TPSG1 | 1 |
| TNIP2 | 1 |
| TM7SF2 | 1 |
| STRADA | 1 |
| SSBP3 | 1 |
| SOWAHC | 1 |
| S100A2 | 1 |
| RUNDC3A | 1 |
| RASIP1 | 1 |
| PWWP2B | 1 |
| PTH1R | 1 |
| PRKAB2 | 1 |
| PIK3R2 | 1 |
| OBSL1 | 1 |
| NFKBIA | 1 |
| NFKB2 | 1 |
| NFATC4 | 1 |
| MSI1 | 1 |
| MICALL2 | 1 |
| MGLL | 1 |
| MEX3A | 1 |
| LMO1 | 1 |
| L1CAM | 1 |
| KIF7 | 1 |
| KCNJ4 | 1 |
| ITPKC | 1 |
| IFNGR2 | 1 |
| IFI30 | 1 |
| ICMT | 1 |
| GPR161 | 1 |
| GPR153 | 1 |
| GNG3 | 1 |
| GLI2 | 1 |
| FOXF1 | 1 |
| FBXO31 | 1 |
| FAM167A | 1 |
| EPHB2 | 1 |
| EPHA2 | 1 |
| ELN | 1 |
| ELF3 | 1 |
| CYP27C1 | 1 |
| CHRDL2 | 1 |
| CERK | 1 |
| CEP68 | 1 |
| CCND1 | 1 |
| BOK | 1 |
| BCL3 | 1 |
| ARHGEF3 | 1 |
| ALS2CL | 1 |
| ALDH4A1 | 1 |

**Supplementary Table 4 Interacting gene counts of identified enhancers**

| Chromosome | Start | End | Interacting gene counts |
| --- | --- | --- | --- |
| chr17 | 81998064 | 82017883 | 7 |
| chr17 | 81990631 | 81996270 | 6 |
| chr8 | 144270409 | 144281724 | 3 |
| chr20 | 62823246 | 62833172 | 3 |
| chr7 | 1476389 | 1488610 | 2 |
| chr3 | 50515081 | 50518817 | 2 |
| chr22 | 30650282 | 30653434 | 2 |
| chr20 | 62627312 | 62629572 | 2 |
| chr16 | 1162162 | 1183908 | 2 |
| chr11 | 2087700 | 2098933 | 2 |
| chr1 | 3473655 | 3477844 | 2 |
| chr1 | 16620239 | 16622471 | 2 |
| chr1 | 153525208 | 153527249 | 2 |
| chr9 | 137312359 | 137321124 | 1 |
| chr9 | 136575805 | 136587171 | 1 |
| chr9 | 134452823 | 134467038 | 1 |
| chr9 | 134429485 | 134439692 | 1 |
| chr9 | 134410798 | 134421370 | 1 |
| chr9 | 134356052 | 134361156 | 1 |
| chr9 | 134208530 | 134215089 | 1 |
| chr7 | 74012921 | 74015256 | 1 |
| chr7 | 2658517 | 2666474 | 1 |
| chr7 | 2643728 | 2650353 | 1 |
| chr7 | 2450072 | 2452876 | 1 |
| chr7 | 1063808 | 1069014 | 1 |
| chr5 | 177452906 | 177457498 | 1 |
| chr5 | 151036052 | 151039695 | 1 |
| chr3 | 56782631 | 56785097 | 1 |
| chr3 | 50382493 | 50396368 | 1 |
| chr3 | 14451177 | 14454443 | 1 |
| chr3 | 14435827 | 14437704 | 1 |
| chr3 | 13503865 | 13510703 | 1 |
| chr3 | 13213629 | 13217240 | 1 |
| chr22 | 50274386 | 50276688 | 1 |
| chr22 | 28950081 | 28954244 | 1 |
| chr22 | 28828500 | 28830270 | 1 |
| chr22 | 19553636 | 19556516 | 1 |
| chr21 | 33396355 | 33398006 | 1 |
| chr2 | 65068535 | 65070384 | 1 |
| chr2 | 127061539 | 127069420 | 1 |
| chr2 | 120771150 | 120774145 | 1 |
| chr2 | 109644071 | 109646743 | 1 |
| chr2 | 10159291 | 10166659 | 1 |
| chr19 | 5126896 | 5136434 | 1 |
| chr19 | 5035321 | 5039834 | 1 |
| chr19 | 48780033 | 48783207 | 1 |
| chr19 | 40661930 | 40664139 | 1 |
| chr19 | 39427567 | 39429447 | 1 |
| chr19 | 1372534 | 1374623 | 1 |
| chr19 | 1359342 | 1361583 | 1 |
| chr19 | 13162943 | 13165955 | 1 |
| chr19 | 1310100 | 1315277 | 1 |
| chr17 | 79799969 | 79802063 | 1 |
| chr17 | 75552079 | 75554077 | 1 |
| chr17 | 42310875 | 42313966 | 1 |
| chr16 | 86497211 | 86502509 | 1 |
| chr16 | 81695459 | 81698549 | 1 |
| chr16 | 1140408 | 1146288 | 1 |
| chr16 | 1034300 | 1041617 | 1 |
| chr15 | 85788446 | 85790009 | 1 |
| chr15 | 85784304 | 85787222 | 1 |
| chr15 | 101119990 | 101122879 | 1 |
| chr14 | 76946902 | 76948923 | 1 |
| chr14 | 35364610 | 35366281 | 1 |
| chr14 | 104180315 | 104185343 | 1 |
| chr11 | 8240809 | 8243366 | 1 |
| chr11 | 74671707 | 74673811 | 1 |
| chr11 | 68016159 | 68018956 | 1 |
| chr11 | 64861591 | 64865356 | 1 |
| chr10 | 97548807 | 97551067 | 1 |
| chr10 | 71073252 | 71077929 | 1 |
| chr10 | 132604804 | 132613775 | 1 |
| chr10 | 132513297 | 132524308 | 1 |
| chr10 | 132384687 | 132389072 | 1 |
| chr10 | 101569982 | 101572060 | 1 |
| chr1 | 6328167 | 6334008 | 1 |
| chr1 | 6158957 | 6161743 | 1 |
| chr1 | 6080708 | 6084591 | 1 |
| chr1 | 54487628 | 54490948 | 1 |
| chr1 | 3479827 | 3483920 | 1 |
| chr1 | 3305441 | 3307767 | 1 |
| chr1 | 3099615 | 3109236 | 1 |
| chr1 | 2441551 | 2447979 | 1 |
| chr1 | 2311830 | 2324890 | 1 |
| chr1 | 22825075 | 22828083 | 1 |
| chr1 | 21299513 | 21303888 | 1 |
| chr1 | 18936983 | 18942278 | 1 |
| chr1 | 16947592 | 16949622 | 1 |
| chr1 | 150536258 | 150538425 | 1 |
| chr1 | 10731631 | 10736024 | 1 |
| chr1 | 10699511 | 10707140 | 1 |
